# Practical Points in the Administration of Ether

**Published:** 1890-03

**Authors:** 


### PRACTICAL POINTS IN THE ADMINISTRATION OF ETHER.

Dr. George F. Shrady, in *Medical Record*, February 23d, 1889, concludes a paper on this subject as follows:

1. In commencing the administration of ether the gradual method is to be preferred.

2. Its employment allows tne lungs to empty themselves of residual air, prevents coughing and struggling, and places the organs in the best possible condition to receive and rapidly utilize the ether vapor.

3. After the stage of primary anæsthesia is reached the more pure ether vapor the patient breathes the better.

4. The shorter the time of anæsthesia, and the smaller the amount of ether used, the less likely are the unpleasant sequelæ to occur.

5. The more evenly it is administered the less shock to the patient.

6. Anæsthesia should be entrusted to experienced administrators only.

7. Many of the fashionable efforts to resusticate patients are not only useless, but harmful.

8. The minimum amount of force should be employed to restrain the muscular movements of the patient.

9. Mixed narcosis is often advisable for prolonged operations.

10. The utility of the galvanic battery, in threatened death, is yet to be proven.

11. The most trustworthy means of resuscitating desperate cases are artificial respiration, hypodermic stimulation, inhalation of nitrate of amyl, and inversion of the body.—*Omaha Clinic.*

